# *Mycobacterium haemophilum* scleritis: two case reports and review of literature

**DOI:** 10.1186/s12886-020-01649-w

**Published:** 2020-09-23

**Authors:** Punyanuch Pisitpayat, Tasanee Sirikul, Poonpilas Hongmanee, Pitak Santanirand, Kaevalin Lekhanont

**Affiliations:** 1 Department of Ophthalmology, Faculty of Medicine Ramathibodi Hospital, Mahidol University, Rama VI Rd., Rajathevi, Bangkok, 10400 Thailand; 2 Department of Pathology, Faculty of Medicine Ramathibodi Hospital, Mahidol University, Bangkok, Thailand

**Keywords:** Nontuberculous mycobacteria, *Mycobacterium haemophilum*, Scleritis, Keratitis, Radial keratoneuritis, Case report

## Abstract

**Background:**

*Mycobacterium haemophilum* is a rare and emerging nontuberculous mycobacteria (NTM). It normally causes localized or disseminated systemic diseases, particularly skin infections and arthritis in severely immunocompromised patients. There have been 5 cases of *M. haemophilum* ocular infections reported in the literature. Only 1 case presented with scleritis with keratitis. Here, we reported 2 cases of *M. haemophilum* scleritis. One of them was immunocompetent host and had keratitis with radial keratoneuritis as a presenting sign.

**Case presentation:**

**Case 1:** A 52-year-old Thai female with rheumatoid arthritis presented with scleritis. Conjunctival scraping was carried out and the culture result was positive for *M. haemophilum*. Despite receiving systemic and topical antibiotics, her clinical symptoms and signs worsened. Surgical debridement was performed. After surgery, the lesion was significantly improved and finally turned to conjunctival scarring. **Case 2:** A 32-year old healthy Thai male without underlying disease presented with nodular scleritis and keratouveitis with multiple radial keratoneuritis. Surgical debridement of the scleral nodule was performed. Initial microbiological investigations were negative. Herpes ocular infections was suspected. Topical antibiotics, oral acyclovir, low-dose topical steroids and systemic steroids were started. The scleral inflammation subsided but later the keratitis relapsed, requiring corneal biopsy. Histopathology of the specimen revealed acid-fast bacteria and *M. haemophilum* was identified by polymerase chain reaction (PCR) and sequencing. The diagnosis of Mycobacterial keratitis was made. Although using the combination of systemic and topical antibiotics, his clinical status progressively deteriorated. Multiple therapeutic penetrating keratoplasties were required to eradicate the infection. No recurrence was found during the 1-year follow-up in both cases.

**Conclusions:**

*M. haemophilum* can cause scleritis and keratitis, even in immunocompenent host. Radial keraoneuritis is first described in *M. haemophilum* keratitis. NTM keratitis should be considered in the differential diagnosis of patients with radial keratoneuritis. Increased awareness and early diagnosis using appropriate culture conditions and molecular techniques are important for the proper treatment of this infection. Prompt surgical intervention appears to be vital for successful management of *M. haemophilum* scleritis and keratitis.

## Background

Nontuberculous mycobacteria (NTM), also known as atypical mycobacteria or environmental mycobacteria, are aerobic, non-motile, non-spore forming bacilli that are naturally found in water, soil, food, and air [1]. Although ophthalmic infections from these opportunistic pathogens are infrequent, its prevalence and variety have been increasing over the past few decades [2]. The highest number of recent clinical reported on NTM ophthalmic infections are of keratitis, followed by endophthalmitis, cutaneous periocular infection, scleritis, dacryocystitis and canaliculitis, orbital infection, uveitis, and conjunctivitis respectively [2, 3]. Most are caused by rapidly growing mycobacteria (Runyon group IV), particularly *Mycobacterium chelonae, M. abscessus, and M. fortuitum groups* [2, 3]. However, slowly growing mycobacteria (Runyon groups I-III) such as *M. avium complex, M. szulgai, M. avium, M. gordonae, M. haemophilum, M. kansasii, M. flavescens, M. marinum, M. nonchromogenicum, M. triviale, M. asiaticum* or NTM of unknown species have also been reported to infect human eyes [2, 3].

*M. haemophilum* is a slow grower and traditionally classified as Runyon group III. It mainly causes cutaneous and subcutaneous infections, septic arthritis, osteomyelitis, pneumonia, and disseminated infection in immunocompromised hosts. Cervicofacial lymphadenitis is another common manifestation in immunocompetent children [4]. This organism rarely affects the eye. There have only been 5 cases of *M. haemophilum* ophthalmic infections reported in the literature. Two cases presented with endophthalmitis [5, 6], 1 case presented with scleritis with keratitis [7], 1 case presented with filamentary keratopathy and conjunctival mass [8], and 1 case presented with dacryocystitis [9]. Here, we reported 2 cases of *M. haemophilum* scleritis. One of them also had keratitis with radial keratoneuritis as a presenting sign. Written informed consent was obtained from both patients to report their clinical data.

## Case presentation

### Case 1

**Fig. 1:**
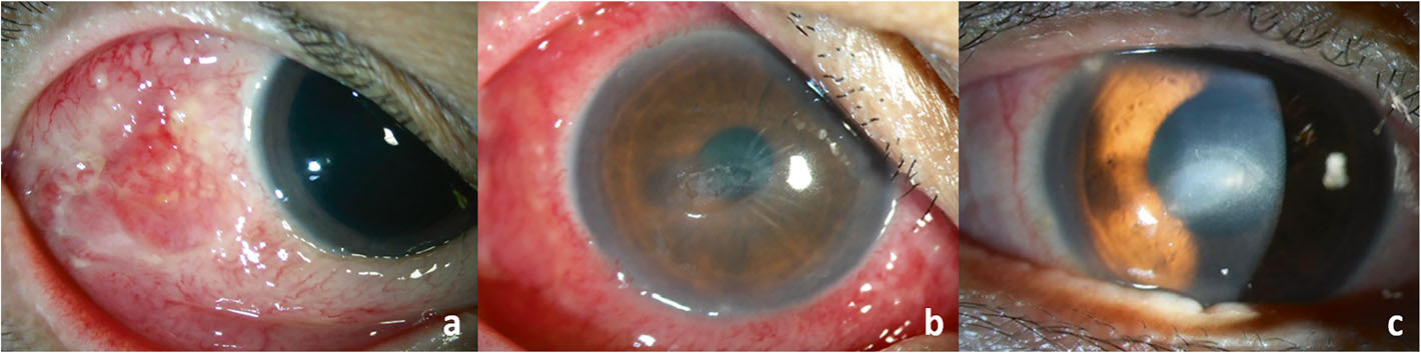
Slit-lamp photography of the right eye at initial presentation shows multiple conjunctival pustules and severe scleral inflammation with overlying conjunctival epithelial defects, extending temporally from the 8 to 10 o’clock position (**a**). Two days after anti-nontuberculous mycobacterial treatment, central corneal epithelial defect without infiltration and diffuse conjunctival hyperemia developed (**b**). After the resolution of the infection, conjunctival and thinned corneal scar were left (**c**)

A 52-year-old Thai female who presented with redness and irritation in her right eye for 1 month was referred to our hospital after 1 week of administration of topical ofloxacin and a combination of chloramphenicol and dexamethasone eye drops without remission. She had underlying rheumatoid arthritis which was well controlled with tofacitinib, methotrexate, and naproxen. She did not have any history of previous ocular trauma or surgery. Her best corrected visual acuity (BCVA) was 20/20 in both eyes. Slit-lamp biomicroscopy revealed multiple conjunctival pustules and severe, sectorial scleral inflammation with overlying conjunctival epithelial defects, extending from the 8 to 10 o’clock position and minimal punctate epithelial erosions at the inferior cornea (Fig. 1a). There was no anterior chamber or vitreous reaction. Fundus examination was unremarkable. B-scan ultrasonography showed no evidence of posterior scleritis. Left eye was normal except for minimal punctate epithelial erosions similar to those observed in the right eye. A diagnosis of infectious scleritis was clinically made.

Conjunctival scraping was carried out. Pus collected from the lesion was stained with Gram stain, 10% potassium hydroxide (KOH), acid-fast and modified acid-fast stains. It was also cultured on blood agar, chocolate agar, Sabouraud’s dextrose agar, thioglycollate broth, Löwenstein-Jensen (LJ) medium and Mycobacteria Growth Indicator Tube (MGIT, Becton Dickinson, Heidelberg, Germany). The specimens were also submitted for polymerase chain reaction (PCR) testing. The smears demonstrated no organism. Empirical treatment was started with topical 0.5% moxifloxacin eye drops 2 hourly and oral moxifloxacin (400 mg) once daily.

The clinical picture did not improve over the following 2 weeks and the initial cultures were all negative. New and larger pustules appeared in the same area. Surgical debridement consisting of conjunctival dissection, de-roofing the pustular lesions and scraping the scleral base of the lesions was performed. Postoperatively, topical 5% amikacin and 0.5% moxifloxacin eye drops were given every hour.

Three weeks later, mycobacterial isolate was detected in standard MGIT. The presence or absence of *M. tuberculosis* complex was evaluated using the *M. tuberculosis* complex specific antigen test (BD MGIT TBc Identification Test, Beckton Dickinson, Sparks, MD, USA) [10]. When negative for *M. tuberculosis* complex, the mycobacterial strain was identified using pan-mycobacterial 16S rRNA gene PCR [10]. At the same time, universal primers targeted to the 16S rRNA gene were used for the optimizing of real-time PCR assay [11]. After the results showed NTM PCR positive, amplified DNA fragments were commercially sequenced from Macrogen Inc., South Korea using ABI 3730XL sequencers. The sequence data obtained were analyzed using the software program BioEdit 7.0.9 [11]. The results were compared with the sequences from the GenBank database, confirming *M. haemophilum.* In vitro antimicrobial susceptibility testing demonstrated that the isolate was sensitive to multiple antimicrobial agents, such as amikacin, rifabutin, rifampicin, clarithromycin, ciprofloxacin, moxifloxacin, and linezolid; and had intermediate susceptibility to doxycycline. The patient was placed on systemic antibiotics including oral rifampicin, clarithromycin, ciprofloxacin, and amikacin; and topical antibiotics including 0.5% moxifloxacin, 5% amikacin and 1.5% azithromycin. Oral tofacitinib and methotrexate were replaced with hydroxychloroquine for her underlying rheumatoid arthritis to minimize the adverse effects of immunosuppression.

Two days after intensive anti-nontuberculous mycobacterial treatment, markedly diffuse conjunctival hyperemia and edema as well as central corneal epithelial defect without infiltration developed (Fig. 1b). Toxic medicamentosa was diagnosed. Topical amikacin and azithromycin eye drops were stopped and topical lubricants were given. After the continuation of systemic antibiotics and topical moxifloxacin eye drops for 6 weeks, the conjunctival pustules and scleral inflammation resolved. Only conjunctival and thinned corneal scar were seen (Fig. 1c). Her BCVA was unaffected. No recurrence within the first year of follow-up was detected.

### Case 2

**Fig. 2:**
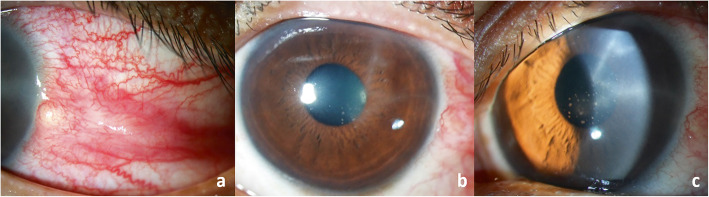
Slit-lamp photography of the left eye at initial presentation demonstrates conjunctival congestion with a 2 × 2 mm scleral nodule in the temporal quadrant adjacent to the limbus, with dilatation of the superficial and deep episcleral vessels (**a**). Few radial keratoneuritis in the upper temporal cornea with intact intervening stroma and overlying epithelium were observed. Some small to medium-sized, non-pigmented keratic precipitates (KPs) were present centrally (**b**-**c**)

A 32-year old healthy Thai male presented with pain and redness in the left eye for 5 weeks. He denied any history of previous ocular trauma, surgery, or contact lens wear. His BCVA was 20/20 in both eyes. Intraocular pressure (IOP) was 15 mmHg in the right eye but increased to 41 mmHg in the left eye. Slit-lamp biomicroscopy of the left eye demonstrated conjunctival congestion with a 2 × 2 mm scleral nodule in the temporal quadrant adjacent to the limbus, with dilatation of the superficial and deep episcleral vessels (Fig. 2a). The nodule was firm, immobile, and tender to palpation. Few radial keratoneuritis in the upper temporal cornea with intact intervening stroma and overlying epithelium were observed (Fig. 2b-c). There was 3+ anterior chamber reaction [12] with some small to medium-sized, non-pigmented keratic precipitates (KPs) centrally (Fig. 2b-c). The rest of the ocular examination was unremarkable. Confocal microscopy showed enlarge corneal nerves, but no cysts or obvious abnormalities were seen. Clinical examination of the right eye was normal. Infectious nodular scleritis with keratouveitis was suspected. Surgical debridement of the scleral nodule was performed. The specimens were stained with Gram staining, KOH, acid-fast and modified acid-fast stains and plated on blood agar, chocolate agar, Sabouraud’s dextrose agar and thioglycollate broth. The smears revealed no organism. The patient was placed on a regimen of 0.5% moxifloxacin eye drops 2 hourly and topical IOP-lowering drugs.

Systemic examination and laboratory work-up for autoimmune diseases, including complete blood count, C-reactive protein, serum electrolytes, renal and hepatic functions, urine analysis, serum rheumatoid factor, cytoplasmic and perinuclear antineutrophilic cytoplasmic antibodies, antinuclear antibodies, angiotensin converting enzyme, syphilitic serology, serology for hepatitis B virus, hepatitis C virus, and human immunodeficiency virus, X-ray of chest and sacroiliac joint showed normal findings.

One week after treatment, there was no significant clinical improvement except for a decrease in IOP. The initial scleral cultures were all negative. Herpes ocular infection was suspected. Topical steroids, oral steroids and oral acyclovir (400 mg) 5 times a day were added. Symptoms and signs gradually improved and all medications were slowly tapered. However, scleral inflammation relapsed after cessation of steroid therapy, once-daily dosing of topical steroids was then maintained.

**Fig. 3:**
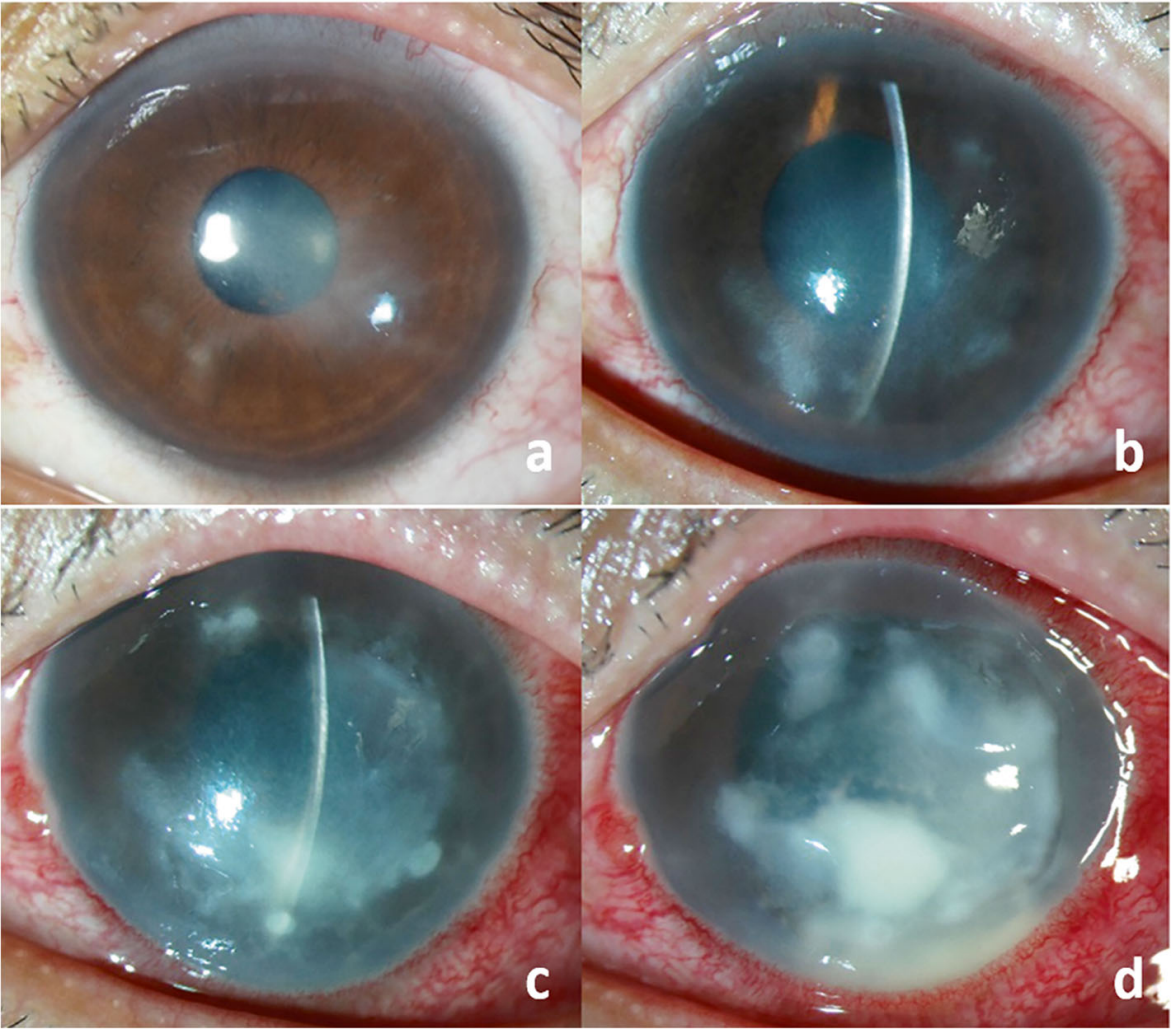
Slit-lamp photography of the left eye at 6 months after presentation shows a relapse with rapid clinical deterioration. Central corneal edema overlying an area of medium-sized, non-pigmented KPs, with some degree of superficial, gray-white, dry stromal infiltrates was present (**a**). One week later, multifocal paracentral stromal infiltrates without epithelial defect developed, along with recurrent radial keratoneuritis and worsening of the anterior chamber inflammation (**b**). Despite aggressive treatment, the corneal lesions became enlarged and rapidly progressed with dense stromal infiltrates and 2 mm hypopyon (c-d), requiring therapeutic penetrating keratoplasty

Six months later, the patient developed central corneal edema overlying an area of medium-sized, non-pigmented KPs, with some degree of superficial, gray-white, dry stromal infiltrates (Fig. 3a). There was 1+ anterior chamber reaction [12]. An aqueous tap was obtained and samples were sent for PCR analysis for human herpes simplex virus type 1 and 2, varicella zoster virus, cytomegalovirus, and *Mycobacterium tuberculosis*. The PCR results were all negative. A presumptive clinical diagnosis of recurrent herpes simplex keratouveitis was made and topical steroids and oral acyclovir therapy were restarted.

One week after treatment, he developed multifocal paracentral stromal infiltrates at varying depths without epithelial defect, along with recurrent radial keratoneuritis and worsening of the anterior chamber inflammation (Fig. 3b). Conjunctival hyperemia was observed with no scleral nodule. Diagnostic corneal biopsy was performed after corneal scraping did not yield positive results and the lesions progressed. Histopathologic examination of the corneal tissue revealed the presence of acid-fast bacilli. Cultures failed to isolate any organisms, but PCR testing identified *M. haemophilum*. Anti-nontuberculous mycobacterial therapy including hourly topical 0.5% moxifloxacin, 5% amikacin and 1.5% azithromycin; and oral rifampin, clarithromycin, amikacin and moxifloxacin were begun.

Despite aggressive treatment, the corneal lesions became enlarged and rapidly progressed with dense stromal infiltrates and 2 mm hypopyon (Fig. 3c-d). Therapeutic penetrating keratoplasty was performed. Regrafting was required twice due to the recurrence of infection in the corneal grafts. Topical and systemic antibiotics were continued for 1 year until the lesion completely resolved. He underwent the 4th penetrating keratoplasty 6 months after antibiotic discontinuation to improve his vision. No recurrence was found during the 1-year follow-up. He achieved a final BCVA of 20/70.

## Discussion and conclusions

To date, 15 reports of NTM scleritis have been published in the literature. The infections were caused by *M chelonae* (17)*, M. fortuitum* (8), *M. abscessus* (1), *M. marinum* (1), and *M. haemophilum* (1) [7, 13–26]. *M. haemophilum* ocular infection was first described in 2007 in a man with graft-versus-host disease who presented with filamentary keratopathy and conjunctival mass and another man with post-cardiac transplantation who presented with purulent endophthalmitis [5, 8]. Since then, it has been 3 more case reports of *M. haemophilum* ocular infection. Here, we present 2 cases of *M. haemophilum* scleritis and one of them also had keratitis with radial keratoneuritis as a presenting sign. The details of all 7 cases were summarized in Table 1.

**Table 1:** Summary of the reported cases of *Mycobacterium haemophilum* ocular infection

No.^**[ref]**^	Age/ sex	Underlying disease	Diagnosis	Exposure to topical steroids	Onset (wk)	Medications		Duration of medications	Surgery	Outcome
						Systemic	Topical			
1 [8]	55/ M	GVHD^a^, DM	Dry eye, FK, conjunctival mass	No	8	Moxifloxacin, clarithromycin, valacyclovir, clindamycin	Preservative-free lubricants, N-acetylcysteine 4 times a day	Not reported	Conjunctival biopsy	Cured
2 [5]	66/ M	Post cardiac transplantation^a^	Endophthalmitis	Yes	N/A	Azithromycin, gatifloxacin, doxycycline, rifabutin^b^		10 months	Vitrectomy, Enucleation	Failed
3 [9]	9/ F	Healthy	Dacryocystitis	No	N/A	Clarithromycin, rifampicin	None	Not reported	Extensive excision	Cured
4 [6]	66/ M	DM, HT, DLP, post multiple glaucoma surgeries	Endophthalmitis	Yes	16	Azithromycin, doxycycline, rifampicin	Levofloxacin	12 months	Vitrectomy	Phthisis bulbi
5 [7]	65/ F	DM	Scleritis and keratitis	N/A	N/A	Imipenem, clarithromycin, levofloxacin, rifampin, linezolid^b^		4 months	Enucleation	Failed
6 (case 1)	52 /F	Rheumatoid arthritis^a^	Scleritis	Yes	4	Rifampicin, clarithromycin, ciprofloxacin, amikacin	Moxifloxacin, amikacin, azithromycin	1.5 months	Debridement	Cured
7 (case 2)	32/ M	Healthy	Scleritis and keratitis	Yes	5	Rifampin, clarithromycin, amikacin, moxifloxacin	Moxifloxacin, amikacin, azithromycin	12 months	Debridement, multiple PKs	Cured

^a^ = on immunosuppressants, ^b^ = the mode of application was not stated, *DLP* Dyslipidemia, *DM* Diabetes mellitus, *FK* Filamentary keratopathy, *GVHD* Graft-versus-host disease, *HT* Hypertension, *N/A* Data not available, *PK* Penetrating keratoplasty

Diagnosis of *M. haemophilum* ocular infection is challenging. The majority of patients with this infection had underlying immunocompromised conditions, including diabetes mellitus, use of immunosuppressive agents, and young age [5–9]. However, one of our 2 cases was an immunocompetent host. It is interesting that our cases and most cases of *M. haemophilum* scleritis did not have predisposing surgeries, such as scleral buckling, pterygium excision, cataract surgery, intravitreal injection, and pars plana vitrectomy. Conversely, almost all cases of other NTM scleritis were directly preceded by surgical interventions [2]. Therefore, a high-index of suspicion is needed to diagnose this infection even in immunocompetent patients without history of accidental or surgical ocular trauma.

The insidious onset of *M. haemophilum* scleritis (4 and 5 weeks in our cases) appeared to be similar to that of other NTM scleritis, which generally varied, ranging from 1.5 weeks to 172 months [2, 3]. There was also no notable difference in the clinical features of *M. haemophilum* scleritis compared to other infectious scleritis [2, 3, 27]. Additionally, the identification of *M. haemophilum* is often difficult and delayed because *M. haemophilum* is a slow-growing acid-fast bacilli and requires special culture techniques and media different from those of most pathogenic NTM species [4]. False-negative stains and cultures occasionally occurred such in our second case, possibly due to inadequate specimen or smear preparation, fastidious/nonviable organisms or inappropriate culture conditions [28]. Thus, *M. haemophilum* can be incorrectly identified as other organisms. Furthermore, some cases were misdiagnosed as viral infection or immune-mediated disease, leading to unnecessary use of steroids similarly to our second case [5, 6].

It is nevertheless intriguing that microbiological diagnosis was achieved by routine culture methods for *Mycobacterium* species in Case 1. Essentially, *M. haemophilum* is unique among *Mycobacterium* species owing to its specific growth requirements [29]. It prefers a lower growth temperature (30°- 32 °C) than other NTM (35°- 37 °C) and needs iron supplements such as hemin or ferric ammonium citrate in both liquid and solid media [4, 29]. Accordingly, the growth of *M. haemophilum* in conventional liquid MGIT medium at 35 °C in Case 1 was likely supported by the sufficient iron provided by blood from the biopsied conjunctival tissue [29].

NTM keratitis is more common than NTM scleritis [2, 3]. Typical characteristics include a “cracked windshield” appearance around the edge of the central area of corneal infiltrates, ring infiltrates, satellite lesions, and infectious crystalline keratopathy [2, 3, 27]. Absence of epithelial defect at initial presentation has been found in up to 1/3 of cases [30]. Nonetheless, dendritic epithelial defects with minimal stromal infiltration were also seen in NTM keratitis [2]. *M. haemophilum* keratitis in our second patient presented with radial keratoneuritis which has never been reported in any case of NTM keratitis. Consequently, besides *Acanthamoeba* keratitis, *Pseudomonas* keratitis, fungal keratitis and *Pythium* keratitis [31–33], NTM keratitis should be considered in the differential diagnosis of patients with radial keratoneuritis.

NTM keratitis might extend to or start at the corneoscleral junction, resulting in sclerokeratitis [7, 14, 17, 18]. *M. fortuitum* (3)*, M. chelonae* (1)*, M. marinum* (1), and *M. haemophilum* (1) were responsible for 6 previously reported cases of sclerokeratitis. Four cases were associated with cataract surgery [17, 18]. Meanwhile, in our second case, it was unclear whether the infection spread form sclera onto the cornea or vice versa. Scleritis seemed to be more predominant than keratitis at the beginning and keratitis occurred alone at the relapsing episode. This might be partly due to the surgical debridement done at the sclera at the first episode.

Therefore, increased awareness and early diagnosis of this infection are crucial for the proper treatment. Despite using the suitable culture condition, it may take up to 8 weeks to observe visible growth of *M. haemophilum* [4]. Molecular identification using real-time PCR assay is an alternative which may enable earlier and more reliable diagnosis [4, 9]. Hence, in every suspected case of NTM ocular infection, a 3-step diagnostic regimen for the optimal detection of *M. haemophilum* including acid-fast staining, culturing at two temperatures with and without iron-supplemented media, and molecular detection is suggested [4]. The recommended culture protocol is as follows: 1) standard mycobacterial culturing at 35 °C in liquid MGIT medium and on solid LJ medium; and 2) *M. haemophilum*-specific culturing at 30 °C in a liquid medium using hemin supplementation and on LJ medium supplemented with iron citrate [4]. In addition, failure to isolate a pathogen from clinical specimens with positive acid-fast stains should prompt a targeted search for *M. haemophilum* [4].

The treatment of NTM scleritis and keratitis needs a 2-pronged approach, which is appropriate antibiotic therapy plus judicious surgery to eradicate infection from affected tissues [2, 3]. Because most ocular NTM infections are recalcitrant to treatment and recur after cessation of therapy, long-term treatment with combination therapy with ≥2 medications is suggested. Systemic antibiotics may also be used. Although there is no consensus on the antibiotics of choice for *M. haemophilum*, previous studies showed that slow-growing NTM were susceptible to anti-tuberculosis drugs, whereas rapid-growing NTM were susceptible to macrolides, fluoroquinolones, and aminoglycosides [3]. Recently, a combination of new-generation fluoroquinolones, rifampin, and new macrolides is recommended for treating *M. haemophilum* [5–7]. Topical steroids are contraindicated [3, 30]. Multiple topical and systemic antibiotics; and surgical interventions for both diagnostic and therapeutic purposes were applied in our 2 cases. The prognosis of NTM scleritis and keratitis could be guarded even with proper treatment [2, 3, 30]. Fortunately, due to early surgical treatments, our patients had favorable visual outcomes.

To the best of our knowledge, little information has been published regarding diagnosis and treatment of *M. haemophilum* scleritis and keratitis. These 2 cases highlight the rare but emerging pathogen that can infect the eye in several different ways. This is also the first report of radial keratoneuritis in NTM keratitis. Real-time PCR assay is a valuable tool for the early detection of the organism.

In conclusion, *M. haemophilum* can cause scleritis and keratitis, even in immunocompenent host. Radial keraoneuritis could be observed in NTM keratitis. The diagnosis of *M. haemophilum* ocular infection may be underdiagnosed because of unawareness among clinicians and challenges in identification of the organism in routine laboratories. The clinician’s vigilance must be increased to early recognize and promptly manage this infection. Consultation with a microbiologist and infectious disease specialist is essential to ensure accurate and timely diagnosis and effective treatment. Early surgical intervention appears to be vital for successful management of *M. haemophilum* scleritis and keratitis.

## Data Availability

Not applicable**.**

## Abbreviations

NTM: Nontuberculous mycobacteria
BCVA: Best corrected visual acuity
KOH: Potassium hydroxide
LJ: Löwenstein-jensen
MGIT: Mycobacteria growth indicator tube
IOP: Intraocular pressure
KPs: Keratic precipitates
PCR: Polymerase chain reaction

## References

[1] van Ingen J, Boeree MJ, Dekhuijzen PN, van Soolingen D (2009). Environmental sources of rapid growing nontuberculous mycobacteria causing disease in humans. Clin Microbiol Infect.

[2] Kheir WJ, Sheheitli H, Fattah MA, Hamam RN (2015). Nontuberculous mycobacterial ocular infections: a systematic review of the literature. Biomed Res Int.

[3] Moorthy RS, Valluri S, Rao NA (2012). Nontuberculous mycobacterial ocular and adnexal infections. Surv Ophthalmol.

[4] Lindeboom JA, van Coppenraet LE, van Soolingen D, Prins JM, Kuijper EJ (2011). Clinical manifestations, diagnosis, and treatment of *Mycobacterium haemophilum* infections. Clin Microbiol Rev.

[5] Modi D, Pyatetsky D, Edward DP, Ulanski LJ, Pursell KJ, Tessler HH (2007). *Mycobacterium haemophilum*: a rare cause of endophthalmitis. Retina.

[6] Pinitpuwadol W, Sarunket S, Boonsopon S, Tesavibul N, Choopong P (2018). Late-onset postoperative *Mycobacterium haemophilum* endophthalmitis masquerading as inflammatory uveitis: a case report. BMC Infect Dis.

[7] Nookeu P, Angkasekwinai N, Foongladda S, Phoompoung P (2019). Clinical characteristics and treatment outcomes for patients infected with *Mycobacterium haemophilum*. Emerg Infect Dis.

[8] Millar MJ, Bulliard C, Balachandran C, Maloof AJ (2007). *Mycobacterium hemophilum* infection presenting as filamentary keratopathy in an immunocompromised adult. Cornea.

[9] Zuercher B, Waridel F, Monnier P, Cherpillod J (2011). A case of dacryocystitis due to *M. haemophilum*. Int J Pediatric Otorhinolaryngol Extra.

[10] Simon A, Onya O, Mazza-Stalder J, Nicod L, Gilbert G, Katia J (2019). Added diagnostic value of 16S rRNA gene pan-mycobacterial PCR for nontuberculous mycobacterial infections: a 10-year retrospective study. Eur J Clin Microbiol Infect Dis.

[11] Keerthirathne TP, Magana-Arachchi DN, Madegedara D, Sooriyapathirana SS (2016). Real time PCR for the rapid identification and drug susceptibility of mycobacteria present in bronchial washings. BMC Infect Dis.

[12] Jabs DA, Nussenblatt RB, Rosenbaum JT, Standardization of uveitis nomenclature (SUN) working group (2005). Standardization of uveitis nomenclature for reporting clinical data Results of the First International Workshop. Am J Ophthalmol.

[13] Pope J, Sternberg P, McLane NJ, Potts DW, Stulting RD (1989). *Mycobacterium chelonae* scleral abscess after removal of a scleral buckle. Am J Ophthalmol.

[14] Schönherr U, Naumann GO, Lang GK, Bialasiewicz AA (1989). Sclerokeratitis caused by *Mycobacterium marinum*. Am J Ophthalmol.

[15] Holland SP, Pulido JS, Miller D, Ellis B, Alfonso E, Scott M (1991). Biofilm and scleral buckle-associated infections. A mechanism for persistence. Ophthalmology.

[16] Smiddy WE, Miller D, Flynn HW (1991). Scleral buckle infections due to atypical mycobacteria. Retina.

[17] Bullington RH, Lanier JD, Font RL (1992). Nontuberculous mycobacterial keratitis: report of two cases and review of the literature. Arch Ophthalmol.

[18] Valenton M (1996). Wound infection after cataract surgery. Jpn J Ophthalmol.

[19] Hsiao CH, Chen JJ, Huang SC, Ma HK, Chen PY, Tsai RJ (1998). Intrascleral dissemination of infectious scleritis following pterygium excision. Br J Ophthalmol.

[20] Margo CE, Pavan PR (2000). *Mycobacterium chelonae* conjunctivitis and scleritis following vitrectomy. Arch Ophthalmol.

[21] Oz O, Lee DH, Smetana SM, Akduman L (2004). A case of infected scleral buckle with *Mycobacterium chelonae* associated with chronic intraocular inflammation. Ocul Immunol Inflamm.

[22] Nielsen JS, Blatt S, Perlman JI, Gieser RG (2004). Clinicopathologic case report: scleral buckle associated nontuberculous mycobacterial scleritis. Semin Ophthalmol.

[23] Liu DT, Lee VY, Chi-Lai L, Lam DS (2007). *Stenotrophomonas maltophilia* and *Mycobacterium chelonae* coinfection of the extraocular scleral buckle explant. Ocul Immunol Inflamm.

[24] Metta H, Corti M, Brunzini R (2008). Disseminated infection due to *Mycobacterium chelonae* with scleritis, spondylodiscitis and spinal epidural abscess. Braz J Infect Dis.

[25] Golen JR, Espana EM, Margo CE. *Mycobacterium abscessus* scleritis following intravitreous injection of bevacizumab. JAMA Ophthalmol. 2013:131 No Pagination Specified. 10.1001/jamaophthalmol.2013.2138. 24151651

[26] Mohan N, Kar S, Padhi TR, Basu S, Sharma S, Das TP (2014). Changing profile of organisms causing scleral buckle infections: a clinico-microbiological case series. Retina.

[27] Garg P (2012). Fungal, mycobacterial, and nocardia infections and the eye: an update. Eye (Lond).

[28] Samuel LP, Balada-Llasat JM, Harrington A, Cavagnolo R (2016). Multicenter assessment of gram stain error rates. J Clin Microbiol.

[29] Sampaio JL, Alves VA, Leão SC, De Magalhães VD, Martino MD, Mendes CM (2002). Mycobacterium haemophilum: emerging or underdiagnosed in Brazil?. Emerg Infect Dis.

[30] Chu HS, Hu FR (2013). Non-tuberculous mycobacterial keratitis. Clin Microbiol Infect.

[31] Roels D, De Craene S, Kestelyn P (2017). Keratoneuritis is not pathognomonic of *Acanthamoeba* keratitis: a case report of Pseudomonas keratitis. Int Ophthalmol.

[32] Kapoor A, Jain R, Sahu S, Sangwan V. Fungal keratitis presenting as radial keratoneuritis. BMJ Case Rep. 2014. 10.1136/bcr-2013-202200. 10.1136/bcr-2013-202200 PMC3948374 24717856

[33] Lekhanont K, Chuckpaiwong V, Chongtrakool P, Aroonroch R, Vongthongsri A (2009). *Pythium insidiosum* keratitis in contact lens wear: a case report. Cornea..

